# Hybrid Rubberised Bitumen from Reactive and Non-Reactive Ethylene Copolymers

**DOI:** 10.3390/polym11121974

**Published:** 2019-11-30

**Authors:** Simona Senise, Virginia Carrera, Antonio Abad Cuadri, Francisco Javier Navarro, Pedro Partal

**Affiliations:** 1 Centro de Tecnología Repsol Ctra. De Extremadura, A-5, km 18, 28935 Móstoles, Spainvirginia.carrera@repsol.com (V.C.); 2 Centro de Investigación en Tecnología de Productos y Procesos Químicos (Pro2TecS), Departamento de Ingeniería Química, Escuela Técnica Superior de Ingeniería, Universidad de Huelva21071, Campus del Carmen, 21007 Huelva, Spain; antonio.cuadri@diq.uhu.es (A.A.C.); partal@uhu.es (P.P.)

**Keywords:** bitumen, tyre rubber, stability, ethylene copolymers, reactive polymers

## Abstract

Hybrid modification is a relatively new concept of incorporating two or more polymeric modifiers of different nature to a bitumen, in order to take advantage of their complementary features. Aiming to this, in this paper, the so-called Hybrid Systems (HSs) were prepared by the addition of an ethylene-based copolymer (reactive or non-reactive) to a model rubberised binder (Crumb Tyre Rubber Modified Bitumen). The resulting binders (referred to as reactive and non-reactivate HSs, depending on copolymer used) were evaluated by means of thermorheological analysis, technological characterisation, fluorescence microscopy and modulated differential scanning calorimetry. From the experimental results, it may be deduced a positive synergistic effect of non-dissolved Crumb Tyre Rubber (CTR) particles and a second polymeric phase that not only improves the in-service performance but also the high-temperature storage stability. This enhancement is attributed to the development of a multiphasic system composed of non-dissolved CTR particles, a polymer-rich phase and an asphaltene-rich phase. In the case of non-reactive HSs, droplets of swollen ethylene copolymer form a well-defined dispersed phase. By contrast, reactive HSs display a different morphology, almost invisible by optical microscopy, related to the development of a chemical network that yields, by far, the highest degree of modification.

## 1. Introduction

The use of Crumb Tyre Rubber (CTR) in road paving applications is not new, since considerable research has been done from the 1960s. However, it still remains a topic of great interest, as witnessed by the recent sharp rise in the number of publications on this matter [1,2,3,4,5,6,7,8,9,10,11,12].

Currently, two distinct technologies for incorporating CTR into road surfacing are being used by road building companies, known as dry and wet processes [1]. A dry process is any method that includes CTR as a substitute for a fraction of the mineral aggregate in the paving mixture, not as part of the binder [1]. By contrast, wet processes employ CTR as a binder additive by hot blending with bitumen to obtain the so-called Crumb Tyre Rubber Modified Bitumen, before being incorporated into asphalt mixture. A typical feature of the latter is that CTR undergoes a notable size reduction and partial digestion during processing, as a consequence of depolymerisation/devulcanization reactions of the three dimensional network of the vulcanised tyre rubber [1,2]. This favours the homogeneity of the rubberised bitumen making wet methods more popular and attractive but also more complex and expensive. However, the degradation of rubber is not 100% complete and, therefore, the final product typically presents a certain amount of non-digested CTR particles, which become further swollen by some light bitumen compounds [2,7]. The presence of these rubber particles has two opposite effects, i.e., an enhancement of the elastic characteristics but also poor storage stability and workability [2,3]. The presently used wet technologies greatly differ in the digestion level of the rubber particles. Hence, for the sake of clarification, wet processes have been classified into two groups, known as the “wet process-high viscosity” and the “wet process-no agitation” or “terminal blend” [1,4]. Thus, “wet process-high viscosity” allows partial digestion of the rubber into the bitumen and leads to a more pronounced modification of in-service properties, due to the presence of a greater number of swollen non-dissolved CTR particles [1,5,6]. Unfortunately, these particles tend to settle when stored at high temperatures and, therefore, these binders require continuous agitation to keep them evenly distributed. By contrast, the terminal blend or “wet process-no agitation” is conceived to yield a high digestion of particles in order to produce a better workability, homogeneity and storage stability, but also poorer high in-service temperature performance [1,9].

Consequently, the existence of swollen rubber particles in the rubberised bitumen contributes to improve the thermomechanical behaviour but leads to problems during storage, road construction and transportation operations. On the one hand, the increased viscosity at working temperatures may cause a poor coating of mineral aggregates and, additionally, the heterogeneity of binder yields interferences between mineral and rubber particles, leading to lay-down and compaction difficulties [2]. On these grounds, the manufacture of rubberised binders in standard plants, with improved homogeneity and hot storage stability, has important practical and economic implications for industry, as an environmentally-friendly and cost-effective alternative to traditional modified bitumens.

To that end, storage stability must be a primary requirement, and efforts must focus on the development of reliable methods for improving this characteristic. The simplest way for rubber stabilization is by using particles fine enough so that friction forces and inter-particle interactions balance gravitational forces [7]. This can be achieved by either using small particles or by an intense processing to ensure a high level of particle digestion, as in the case of the “wet process-no agitation” [1]. However, even though highly digested particles lead to enhanced storage stability, it happens at the expense of in-service properties [1,2]. Therefore, taking into account the difficulty to combine enhanced performance properties with acceptable storage stability, intense research efforts have been focused on that matter [10]. To this end, different methods have been proposed in the literature such as adding chemical modifiers [8], a second polymeric phase [10], sulphur [9], etc.

In this sense, all this previous research raises the possibility of enhancing hot storage stability by promoting interactions of a new polymer-rich phase with non-digested rubber particles, producing the so-called Hybrid Systems, HSs hereinafter [10]. In addition, the joint use of both tyre rubber and a thermoplastic polymer allows to take advantage of their supplementary properties to modify a model bitumen. Thus, CTR contributes to enhance the performance in the whole temperature range, particularly at low temperatures, yielding a notable increase in flexibility, whereas the other polymer mainly affects the high in-service temperature properties, leading to a better rutting resistance of the binder [9,11].

Depending on the type of polymer used, HSs can be classified in two categories: non-reactive or passive HSs (where final composition is formed by physical means) and active or reactive HSs (where chemical reactions happen involving the polymer phase and some bitumen molecules). Some representative examples of the former group are low-density polyethylene [11,12], Styrene butadiene styrene (SBS) [13] and ethylene-vinyl-acetate (EVA), ethylene butyl acrylate (EBA) [10] and of the latter, SBS-glycidylmethacrylate [14] and SBS-sulphur [9,10].

To gain more insight into this matter, this paper presents a comparison of several ethylene copolymers of different natures, reactive and non-reactive, in the formulation of novel compatible Hybrid Systems (HSs) which may be of interest to the industry. In this sense, the main goal of this paper is aimed to enhance both high temperature storage stability and in-service properties through physical and chemical processes, and further interactions of CTR and the secondary polymer phase. To that end, a detailed thermorheological and technological test and microstructural analysis have been performed.

## 2. Materials and Methods

### 2.1. Materials

A rubberized bitumen containing 10 wt % Crumb Tyre Rubber (hereinafter called CTR70) was firstly obtained to serve as a base for the formulation of Hybrid Systems (HSs). After processing, this base binder presents a penetration grade of 70 dmm. For comparative purposes, a neat bitumen of similar penetration (70/100) was also included in this study.

For all systems, a commercial Crumb Tyre Rubber (particles smaller than 0.8 mm sieve size), donated by Repsol S. A. (Spain), was used. Its chemical composition is as follows: 57 wt % total rubber hydrocarbon (natural and synthetic rubber), 30 wt % carbon black, 10 wt %, THF extractable and 3 wt % ash.

Different ethylene based copolymers, provided by Repsol S. A. (Spain), were used to formulate HSs. Melt flow rates, at 230 °C, and polymer composition were gathered in Table 1.

PBEa and PBEb are non-reactive propylene-based elastomers formed by isotactic propylene repeat units with random ethylene blocks, produced using metallocene catalyst technology, and with 11.0 and 15.0 wt % of ethylene content, respectively. EMAa and EMAb are fully saturated ethylene-co-1-octene copolymers functionalized by reactive extrusion with maleic anhydride, containing 0.85 and 0.35 wt % of pendant succinic anhydride groups respectively. Finally, the reactive polyolefin (RPO) is composed of a mixture of isotactic polypropylene, ethylene vinyl acetate copolymer (EVA), polyethylene wax and poly glycidylmethacrylate (5 wt %).

### 2.2. Sample Preparation

Ternary blends, or Hybrid Systems (HSs), were formulated by blending rubberised binder (CTR70) and 3 wt % of these polymers (with the exception of RPO), following the procedure described below. Since the sample formulated with 3 wt % reactive polyolefin (HS-RPO) tends to form a strong gel that cannot be used for paving applications, RPO concentration in HS-RPO was limited to 2 wt %.

Firstly, the base CTR70 was prepared by high shear mixing 10 wt % CTR at 180 °C, with a soft bitumen to achieve a penetration of 70 dmm, at pilot plant scale, using a Dispax-Reactor^®^ type 2000/4-HT from IKA^®^-Werke GmbH (Staufen im Breisgau, Germany). Next, Hybrid Systems (HSs), were obtained by adding the second polymeric modifier, at 180 °C, in a high shear mixer Silverson L4RT (Chesham, UK) for 2 h and, then, cured in an oven at 160 °C for 24 h, in order to complete the chemical reactions.

### 2.3. Tests and Measurements

All modified bitumens were submitted to selected technological tests (penetration, softening point, force ductility, elastic recovery and Fraass breaking point) according to EN 14023 normative, as summarised in Table 2.

The penetration is defined as the distance (in tenth of millimeter, dmm) that a standard needle penetrates into the sample under given conditions of loading. The softening point measures the temperature at which a small steel ball placed on top of a bitumen deflects and sags to a distance of 25 mm, when sample is heated at a controlled rate of 5 °C/min. The elastic recovery is measured by the percentage to which a bituminous sample recovers its original length after it has been elongated 400 mm with a speed of 50 mm/min and then cut in half.

The hot storage stability, at 180 °C, was determined by the difference of softening point temperatures of the top and bottom sections of aluminium toothpaste tubes (32 mm in diameter and 160 mm in height) in accordance to UNE-EN 13399.

The microstructure of the HSs was obtained at ambient temperature by means of fluorescence microscopy following UNE-EN 13632 using a Leica DM 2500 microscope (Leica, Wetzlar, Germany) with a 400X magnification.

Small amplitude oscillatory shear (SAOS) tests were performed to characterise the linear viscoelastic response of the HSs. Thus, frequency sweep tests were performed at constant temperatures (40, 60 and 80 °C), from 0.1 up to 10 Hz using smooth plate-and-plate geometry (25 mm diameter, 1–2 mm gap), at stresses within the linear viscoelasticity region. In addition, temperature dependence was studied by temperature sweep tests applying 1% strain at the selected frequency of 10 rad/s, from 40 to 100 °C at a heating rate of 1 °C min^−1^. Multi Stress Creep Recovery tests (MSCR) were performed, at 60 °C, in accordance with the EN 16659 standard. All rheological tests were done with a Dynamic Shear Rheometer (DSR-Physica MCR302, Anton Paar, Graz, Austria).

Modulated differential scanning calorimetry (MDSC) was performed using a DSC-Q100 calorimeter (TA Instruments, New Castle, Delaware, USA) under N_2_ atmosphere. About 5–10 mg of every sample was hermetically sealed in aluminium pans and heated at 150 °C for 5 min and, then, placed at room temperature for 24 h before measurement to ensure the same thermal history. During the test, samples were firstly cooled down to −80 °C, and held at this temperature for 5 min to temper them, and, then, thermograms were done using an average heating rate of 3 °C/min to 120–200 °C, with an oscillation period of 60 s and amplitude of ±0.50 °C.

## 3. Results and Discussion

### 3.1. Technological Characterization and Storage Stability

In order to make a quick comparison of the performance of all modified bitumens, the results of selected technological tests, according to EN 14023, are presented in Table 3.

By comparing CTR70 with a model neat bitumen of similar penetration range (70/100), the expected positive effects of CTR are revealed: higher softening point (5 °C), improved elasticity (much larger elastic recovery), enhanced low temperature flexibility (reduction of 10 °C in Fraass breaking point) and lower temperature susceptibility (higher penetration index values) [11,12]. Unfortunately, Table 3 also discloses the poor high temperature storage stability of CTR70, as may be easily deduced from the difference of 10 °C between the softening point at the top and bottom sections of the settling tube, which noticeably exceeds the limit of 5 °C established by EN 13399. This well-known phenomenon results from the settling of non-dissolved rubber particles during hot storage and represents one of the main drawbacks that limits the use of rubberised bitumens by the industry [1,7].

**Figure 1:**
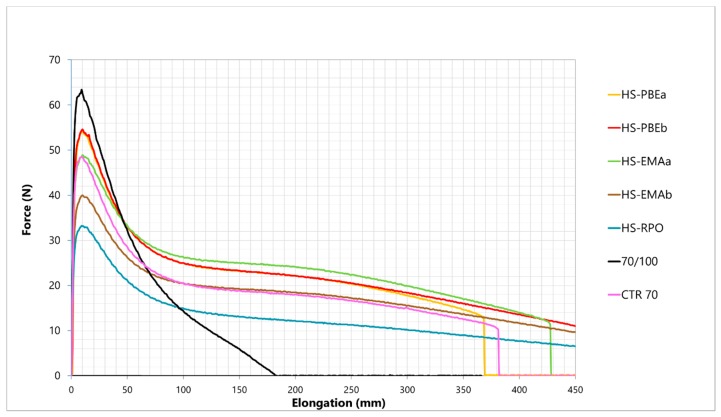
Force ductility curves, at 5 °C, for the studied binders.

In this sense, according to the results presented in Table 3, it is clear that all ethylene copolymers used in this study lead to a notable improvement in hot storage stability in such a way that, with the exception of HS-EMAb, these binders fulfil the stability requirement stabilised by the European standard EN 13399. Furthermore, Table 3 also points out that technological properties such as penetration, softening point and cohesive energy are greatly improved after the addition of the ethylene copolymer, whereas Fraass breaking point and elastic recovery remain almost unaffected. This result is considered satisfactory since low-temperature properties and elastic recovery are often deteriorated after modification with ethylene-based polymers [10,15]. This outcome is in line with force-ductility tests, at 5 °C, reported in Figure 1. Thus, with the exception of HS-PBEa, all HSs binders give rise to elongations larger than the required 400 mm established by EN13703 and, therefore, improve the observed behaviour for the base rubberised bitumen which breaks before reaching this length.

As expected, all systems exhibit a ductile-like behaviour characterised by an early sharp peak (yield point) in the elongation process followed by a smooth decrease during the plastic deformation period until the rupture point. Even though neat bitumen presents the highest maximum at the yield, the force declines sharply and breaks very rapidly after the yield drop. Despite the fact that CTR70 does not strictly fulfil the breaking criteria, its force-ductility behaviour is very close to the hybrid systems and, in addition, its cohesive energy (area below the resulting force-distance function calculated between 200 and 400 mm, Table 3) is clearly above the values typically required for a polymer modified bitumen (at least 2 J·cm^−2^). Therefore, taking into account that remaining CTR particles usually act as crack initiators leading to a premature breakage during elongation [16], this result hints at an enhanced digestion of CTR and, therefore, a better homogeneity of the rubberised bitumen. In general, according to Figure 1 and Table 3, no direct correlation between the type of polymer and force ductility results can be established, although HS-PBEb and HS-EMAa show the best results in terms of stiffness and cohesion energy.

**Figure 2:**
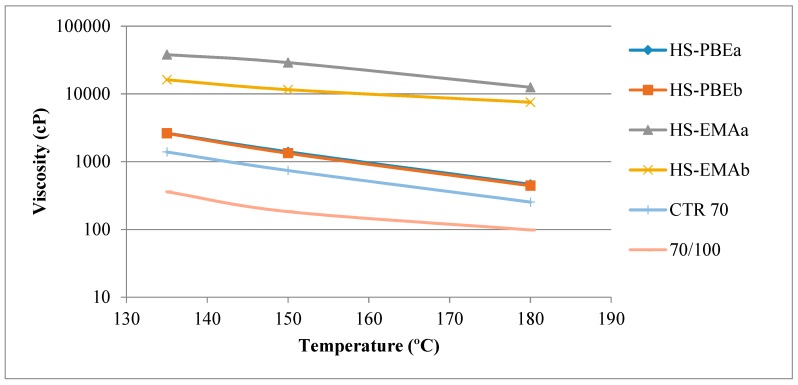
Evolution of Brookfield viscosities with temperature for the studied binders.

Figure 2 gathers Brookfield viscosity data at 135, 150 and 180 °C, which are of a particular interest for mixing and compaction operations.

By comparing neat bitumen with CTR70, it is clear that CTR addition gives rise to an increase in binder viscosity, mainly attributed to the existence of non-dissolved tyre rubber particles [7]. Further polymer modification to form HSs yields an additional increase in viscosity that is dependent on the type of polymer used. Thus, while HS-PBEs only present a slight rise, HS-EMAs undergo a remarkable increase in high temperature viscosities, particularly for HS-EMAa. Therefore, all reactive systems (HS-EMAs) fail to meet the Superpave criterion that requires a maximum viscosity of 3 Pa·s at 135 °C, in order to ensure bitumen fluidity for pumping during delivery and workability in plant operations [12]. This result hints that, as a consequence of chemical processes, EMA polymers develop a polymeric structure able to resist high temperatures. However, since this requirement was developed for Newtonian bitumen, it is not strictly applicable to shear-thinning materials like rubberised bitumen and hence, this limiting viscosity can be exceeded if the bitumen can be pumped and mixed with aggregates [3].

### 3.2. Rheological Characterization

**Figure 3:**
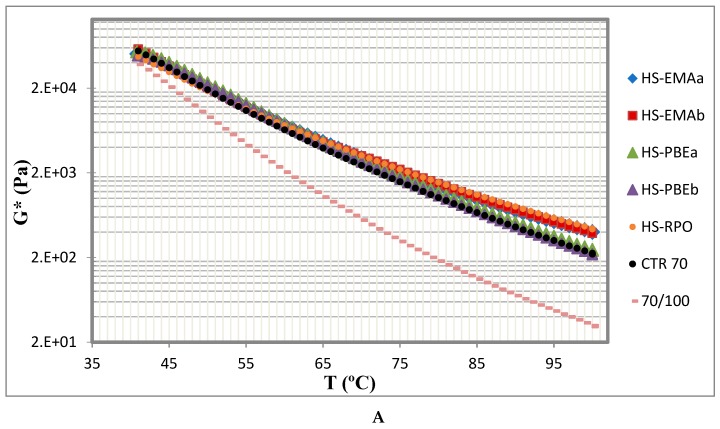

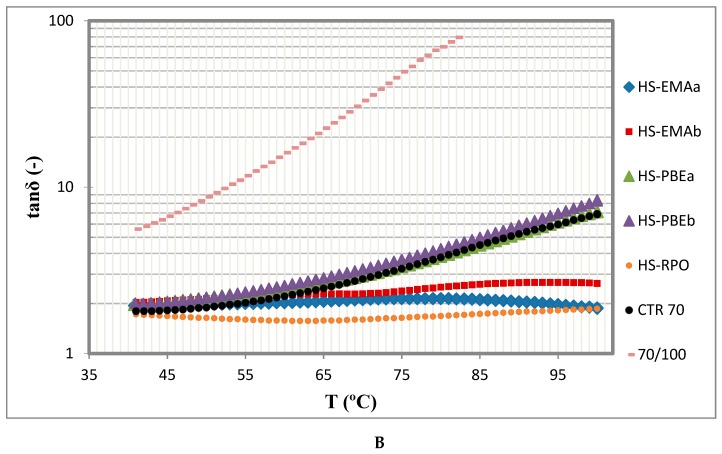
Temperature dependence of (**A**) complex shear modulus and (**B**) loss tangent, at 10 rad/s, for the studied systems.

The effects of rubber and polymeric modifiers on the performance of binders over a wide range of temperatures can be easily analysed by means of the dynamic shear temperature sweeps (Figure 3).

As expected, complex shear modulus undergoes a continuous decrease with testing temperature, from 30 to 100 °C, for all samples (Figure 3A). Hybrid modification causes a remarkable increase in complex shear modulus (G*), more evident as temperature is raised, when compared to the reference neat bitumen. As the complex modulus is correlated with the stiffness of the sample, this result points out a better performance in the intermediate and high in-service temperature range, and presumably enhanced resistance to permanent deformation as well [11,12]. Furthermore, temperature susceptibility is improved, because the average slope of G* and loss tangent (tan*δ*) vs. temperature lowers (Figure 3A,B), within the testing interval. Consequently, this sample shows a better resistance to thermal changes and so the resulting pavement [11].

In addition, as loss tangent presents greater sensitivity to modification changes, Figure 3B may be used to quantify the contribution of every polymer to the global rheological response of the HSs, in the sense that, the lower the values of tan*δ*, the better the modification effect is.

Finally, Figure 3B also allows distinguishing two types of rheological behaviours after polymer addition. On the one hand, CTR70 and HS-PBEs present curves with similar qualitative evolution with temperature than neat bitumen, characterised by a continuous increase of the loss tangent, a prevailing viscous behaviour (tan*δ* > 1) and the absence of the “plateau” region, over the entire temperature interval tested. Hence, a direct transition from the glassy to the Newtonian region is a distinctive characteristic of these non-reactive systems. On the other hand, for reactive systems (HS-EMAs and HS-RPO), loss tangent curves become virtually independent of temperature getting values close to 1, which is considered the rheological response of a critical gel [11].

**Figure 4:**
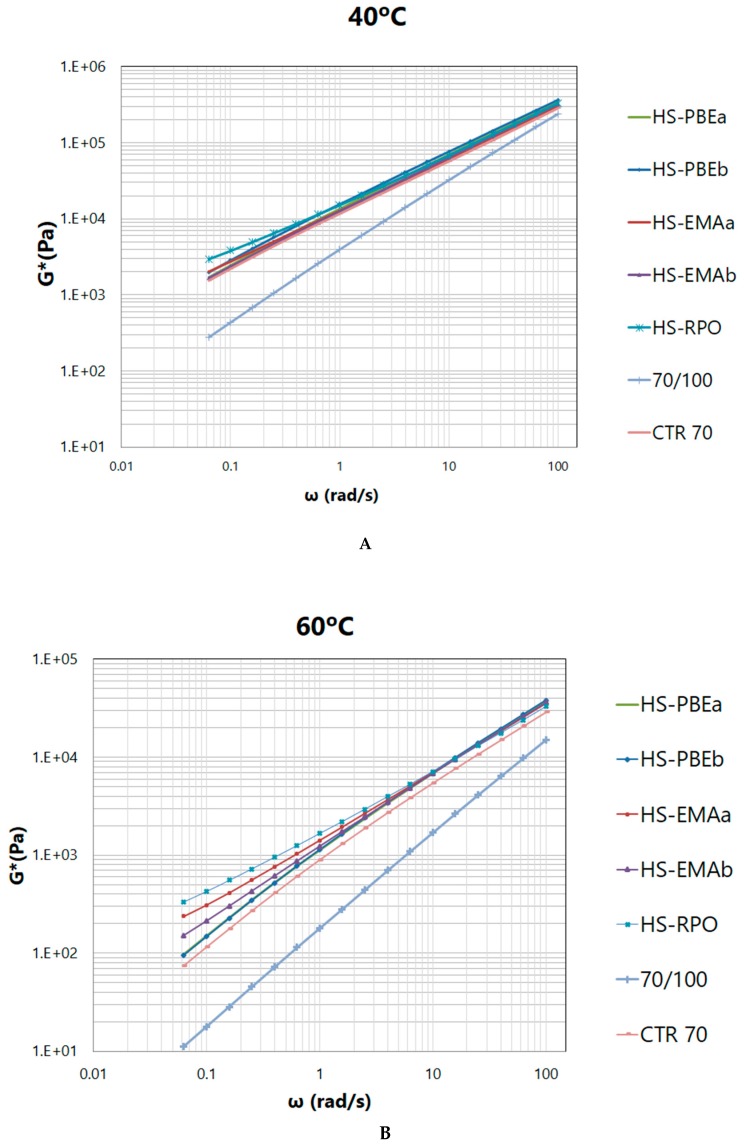

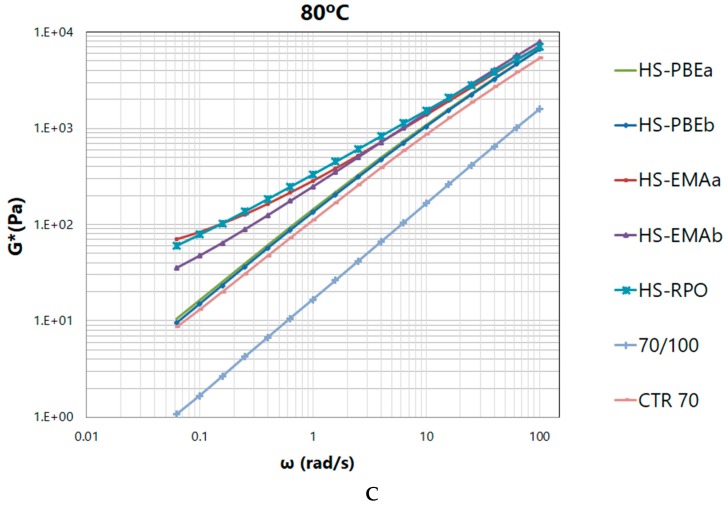
Frequency (ω) dependence of the complex shear modulus, for the studied systems at (**A**) 40 °C, (**B**) 60 °C and (**C**) 80 °C.

The used frequency (10 rad/s) in accordance with Superpave specifications was chosen for performance criteria because it simulates the deformation rate caused by a car travelling at 60 km/h. However, from a rheological point of view, this value is considered relatively high and may mask differences among samples. In this sense, frequency sweep tests, at selected constant temperatures (40, 60 and 80 °C) were also performed in order to provide a deeper insight into structure formed by crumb rubber and polymers. Figure 4 shows the typical evolution of G* of bituminous materials at high temperatures (at 40, 60 and 80 °C), characterised by a steady increase of the complex modulus with frequency.

As testing temperature rises and frequency lowers, differences among samples become more apparent, pointing out the effects caused by ethylene copolymers. It is important to note that, once again, two distinctive behaviours are revealed. Thus, the group of the non-reactive samples (neat bitumen, CTR70 and HS-PBEs) is characterised by a predominant viscous character, which is intensified as temperature rises, showing a trend to reach the flow region of the mechanical spectrum, at low frequencies. On the contrary, reactive HSs undergo a flattening on complex shear modulus curves and develop a plateau in the loss tangent, confirming the previously mentioned critical gel-like behaviour.

**Figure 5:**
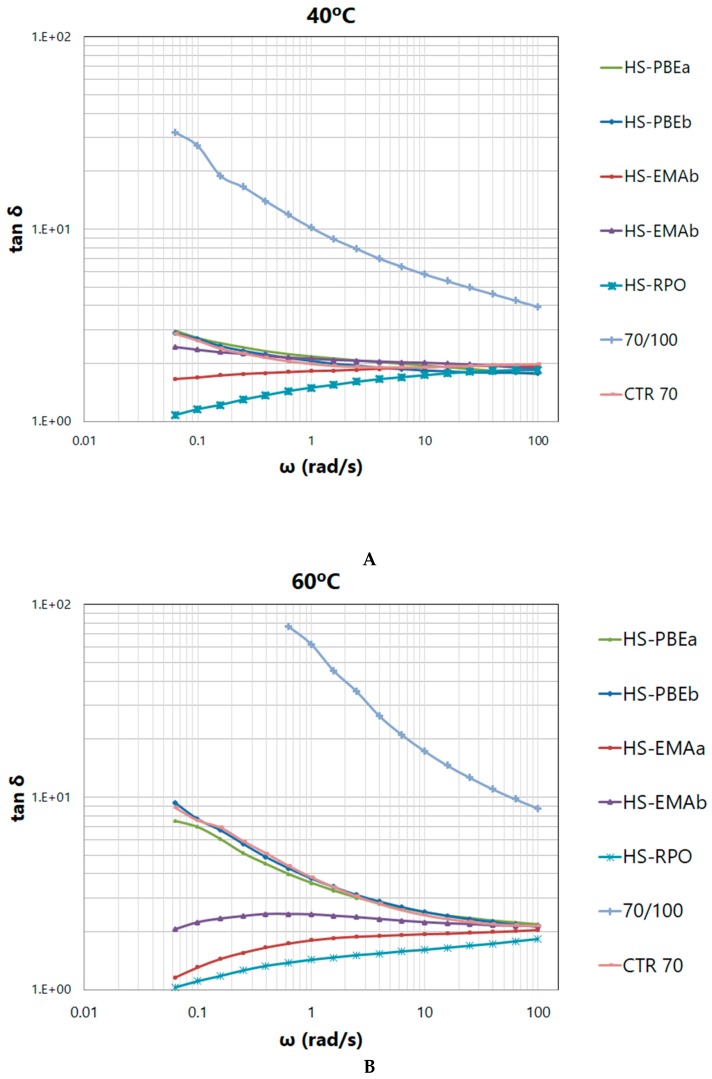

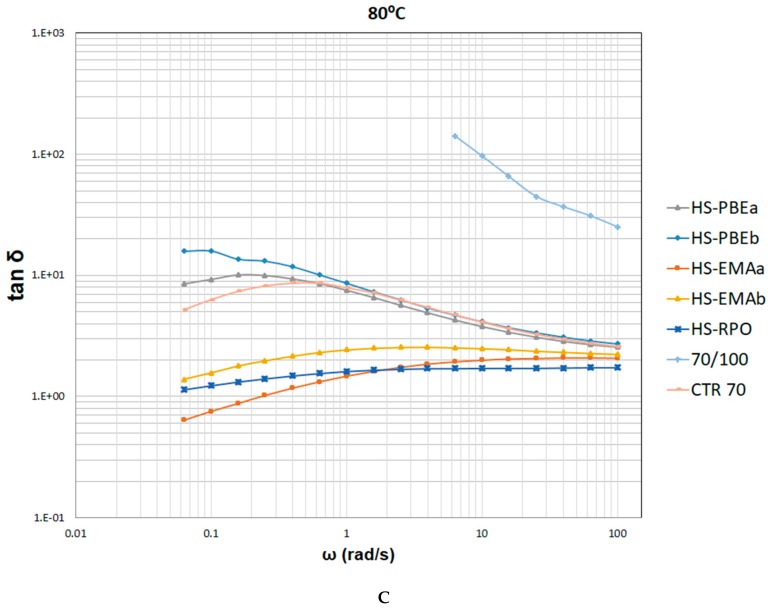
Evolution of the loss tangent with frequency, for the studied systems at (**A**) 40 °C, (**B**) 60 °C and (**C**) 80 °C.

Taking into account that loss tangent is inversely proportional to the overall elasticity of the sample, Figure 5 allows samples to be classified with respect to the modification potential of the polymers used, resulting: PBEb ≈ PBEa << EMAb < EMAa < RPO.

**Figure 6:**
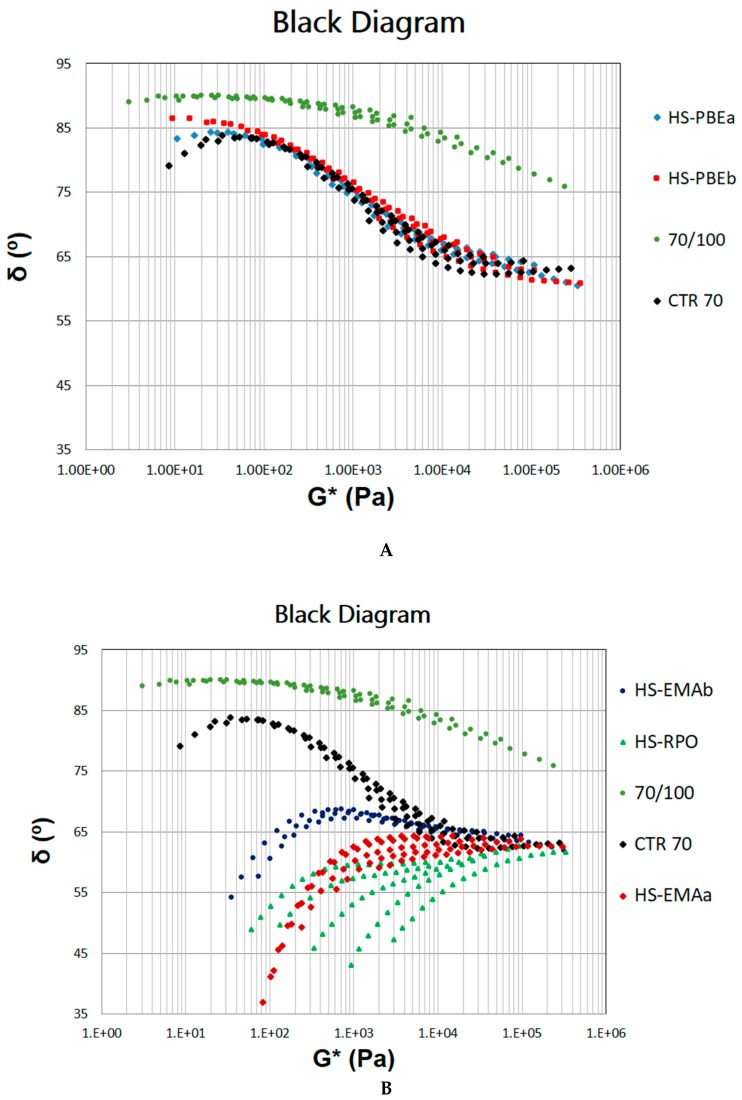
Black Diagrams for (**A**) reactive and (**B**) non-reactive Hybrid Systems (HSs). Neat bitumen (70/100) and the rubberised binder Crumb Tyre Rubber (CTR70) have been included for comparison.

The observed change in the thermomechanical response is more clearly evidenced by the so-called Black diagrams (δ vs. G*, see Figure 6) where non-reactive (A) and reactive HSs (B) are portrayed separately with 70/100 (neat bitumen) and CTR70 as references. These graphs have been widely employed for modified bitumens not only for verifying the time-temperature superposition principle, but also to evaluate structural changes [10].

As may be seen in Figure 6A, whereas neat bitumen presents nicely superposed curves and a transition to a wide Newtonian region (*δ* = 90°), non-reactive systems present curves which are not completely overlapped, with a notable modification of the rheological behaviour. Thus, the phase angle is shifted downward, tending to develop a plateau of the phase angle as G* increases. This fact points out an enhancement in elasticity and is indicative of structural changes in binder microstructure.

Changes in viscoelastic behaviour are much more evident for reactive HSs (Figure 6B) which hardly present a maximum of *δ* in the low G* region, and phase angle values decrease towards 45° (i.e., towards a predominantly elastic behaviour at low frequencies). In addition, as modification is more intense, isothermal curves do not overlap at all, pointing out a thermorheologically complex behaviour. This outcome is attributed to the different temperature dependences of the relaxation times of the expected dispersed phases (non-dissolved rubber particles and polymer-rich phase) [3,10]. Consequently, these results reveal the multiphasic nature of the HSs in which non-dissolved CTR particles and polymer phases play a key role.

It is important to note that HSs present a remarkable improvement in binder elasticity, as may be deduced from the linear viscoelastic behaviour, which is usually related to a better in-service performance. However, taking into account that distresses like pavement rutting take place at stresses larger than those of the lineal viscoelastic range, these functions cannot fully account for the performance characteristics of modified binders and may be inadequate in rating a polymer-modified binder [17].

With this aim, the multiple stress creep recovery test (MSCR, UNE-EN 16659) has been proposed to evaluate how prone to permanent deformations a binder is. MSCR characterises the recovery and non-recovery properties of the bitumen by means of 10 cycles of 1 s creep followed by 9 s recovery, at two consecutive stress levels (0.1 and 3.2 kPa). The chosen temperature was 60 °C because this value is typically considered the maximum expectable pavement temperature in southern European countries. Taking into account that irreversible deformations of asphalt mixes are highly dependent on the stress levels, rutting itself is a non-linear viscoelastic phenomenon and, therefore, the MSCR test is considered a better method in evaluating the rutting resistance.

**Figure 7:**
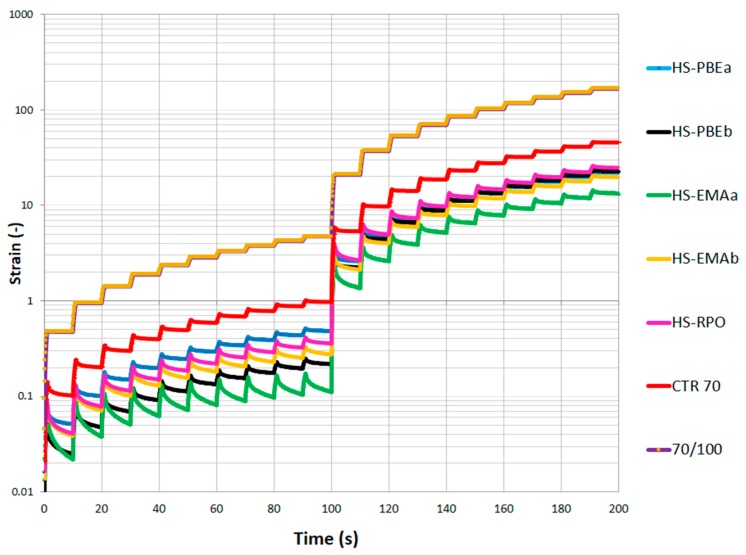
Multiple stress creep recovery test (MSCR) test results performed at stresses of 0.1 (<100 s) and 3.2 kPa (>100 s) for the studied systems.

As shown in Figure 7, neat bitumen undergoes large strains and small magnitudes of strain recoveries, forming a staircase pattern, typical of a viscous Newtonian response.

The improved elasticity previously reported for the rubberised bitumen (CTR70) and, especially for HSs, gives rise to a notable modification in strain profiles on MSCR. Thus, in every cycle, delayed elastic strain recoveries are clearly observed, leading to a remarkable non-linear response, since accumulated compliances become stress dependent [18].

In addition, it is important to underline that strain recoveries results clearly increased, in particular for reactive HSs, in such a way that a sawtooth-shaped profile is disclosed, which is associated to highly modified bitumen with enhanced elastic response. Again, the synergistic effect of non-dissolved CTR particles and the second polymeric phase seems to be the responsibility for the significant change in the creep recovery profile. Regarding the in-service issues, it is noteworthy that the reduced cumulative strains for HSs would lead to an improved rutting resistivity potential of the pavement.

In addition, the MSCR test allows to obtain two important average parameters from every set of 10 cycles, the non-recoverable creep compliance (*J*_nr_) and the percent of elastic recovery (%*R*), which can be employed to characterise the creep response at the selected stress levels (0.1 and 3.2 kPa). The percent of elastic recovery (%*R*) quantifies the elastic character of the material, whereas the non-recoverable compliance (*J*_nr_) measures the quantity of energy dissipated during the recovery runs and, therefore, gives indications of the proneness of asphalt mixtures to undergo permanent deformation damages. Consequently, both parameters enable a quantitative analysis of MSCR results (Table 4).

In general, all reactive HSs and especially HS-EMAa, exhibit the largest %*R* and the lowest *J*_nr_ values in magnitude, at each of the applied test stresses, pointing out a good elastomeric behaviour and a lower rutting susceptibility. In this regard, AASHTO M 332, [19] introduced a new method to classify binders in terms of their elasticity by means of an exponential function of non–recoverable creep compliance, at the cycle of 3.2 kPa:

(1) $$\%R_{3.2kPa}>29.37J_{nr\;3.2kPa}^{-0.263}$$

Therefore, bitumens with a percent of elastic recovery higher than this limit are expected to have good elastomeric behaviour [20]. Consequently, according to this criterion, HS-EMAa and HS-RPO present the best performance under rutting conditions. However, it must be noted that this condition only provides a pass or fail criterion and does not allow a quantitative evaluation, because the calculated compliance parameter is calculated from *J*_nr, 3.2kPa_ which changes from binder to binder [20].

### 3.3. Correlation of Properties with Microstructure: Optical Microscopy

**Figure 8:**
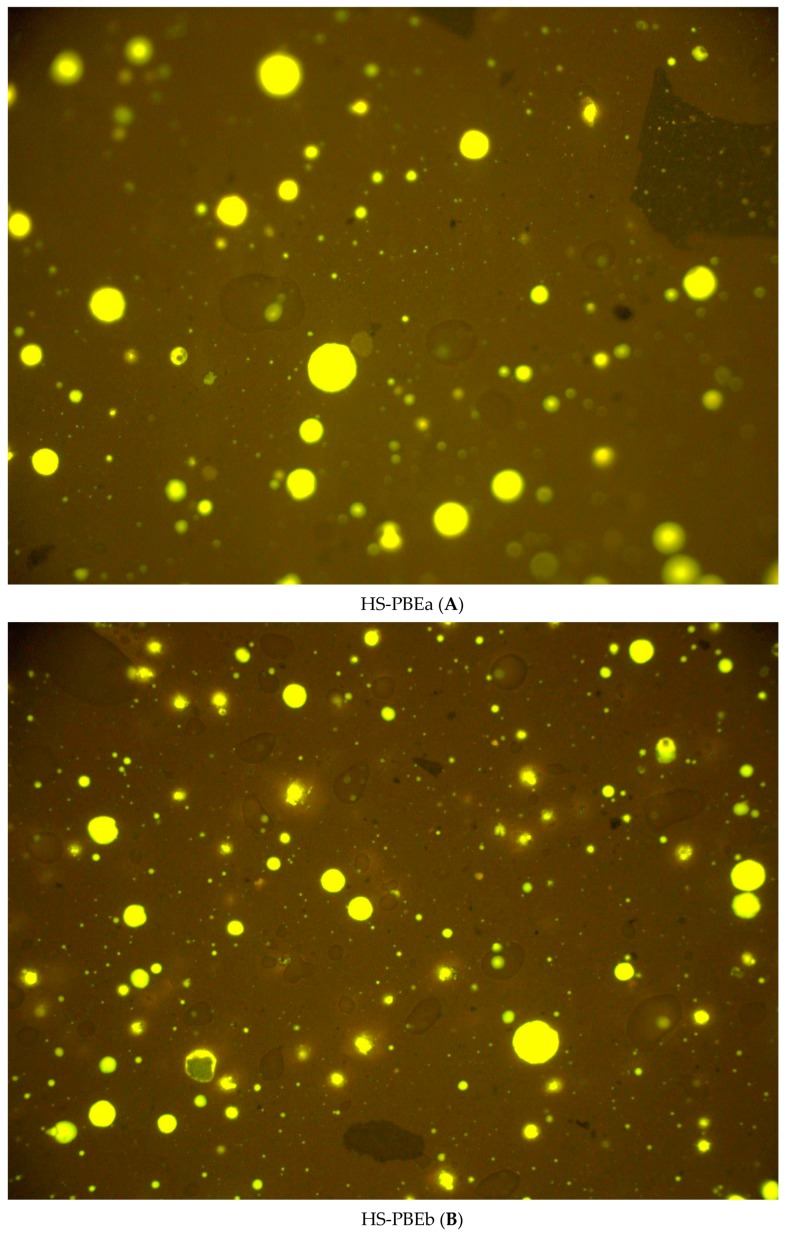

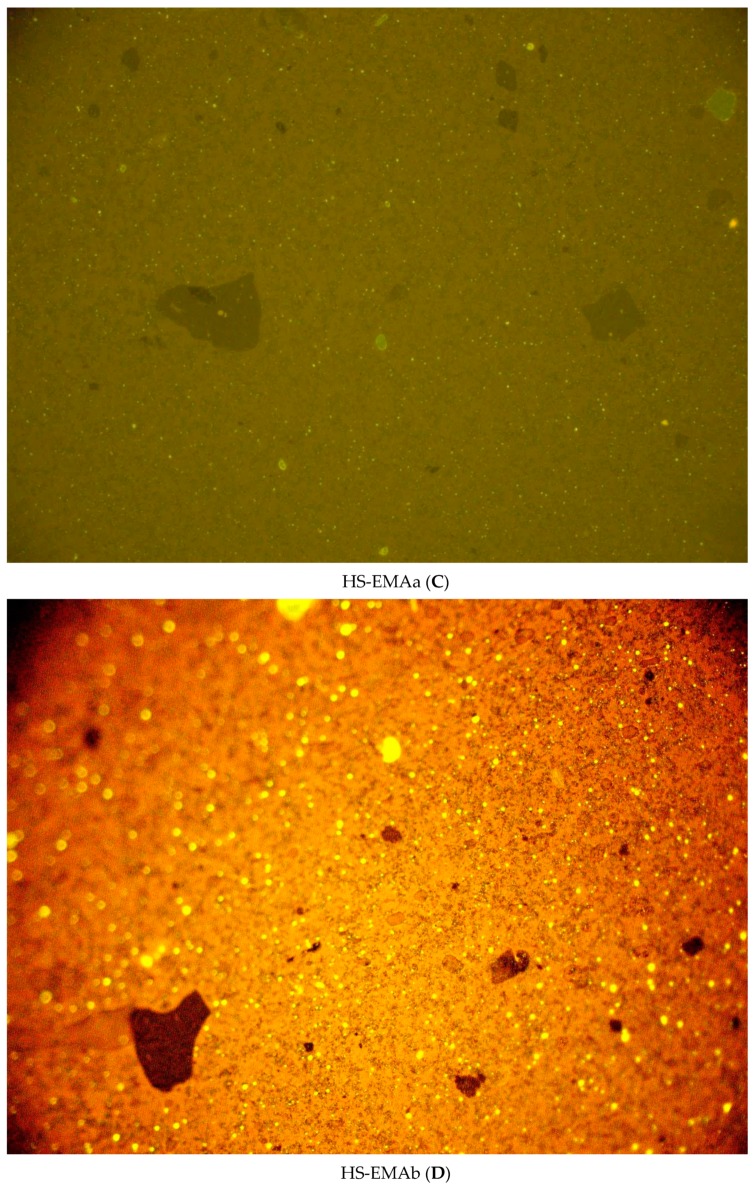

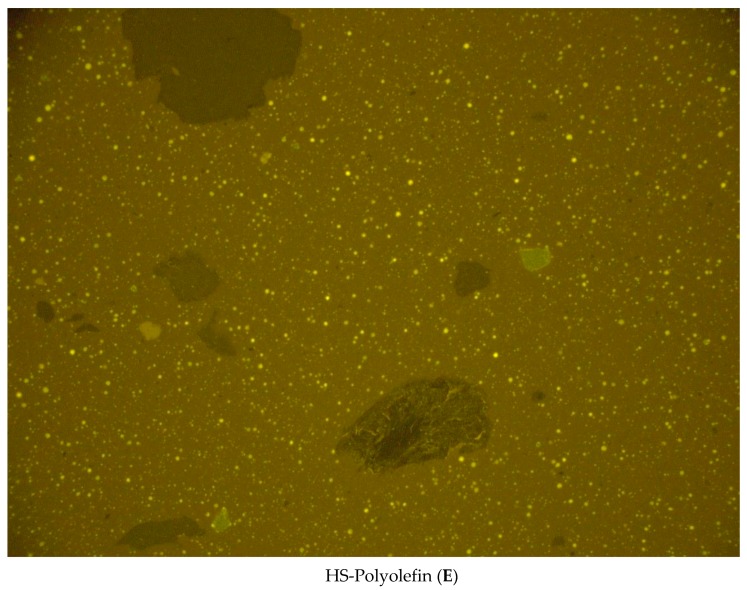
Fluorescence optical microscopy images of Hybrid Systems: (**A**) HS-PBEa, (**B**) HS-PBEb, (**C**) HS-EMAa, (**D**) HS-EMAa, and (**E**) HS-RPO.

From previous results, it is clear that different improvement degrees are found after the modification of the rubberised bitumen, depending on the type of polymer used. The thermomechanical behaviour of HSs could be explained by the development of a complex multiphasic system formed by polymeric phases of a different nature [11], as may be supported by fluorescence microscopy (Figure 8).

On the one hand, all HSs presented in Figure 8 show, as darker colour regions, the presence of non-dissolved CTR particles of different sizes, resulting from the digestion of the original rubber. As it has been widely reported [2,11], these fine elastic particles together with the dissolved rubber chains give rise to the observed improvement for CTR70. It is noteworthy that the presence of these particles, far from acting as cracking promoters of the asphalt, contribute to the improvement of the low-temperature properties, as evidenced by the lower Fraass Point compared to a neat bitumen (Table 3) [11]. In addition, Figure 8 also displays the existence of dispersed light-toned regions, pointing out the presence of a polymer-rich phase for all HSs. However, different proportions and size distributions of the polymer-rich phases are clearly distinguished. On the one hand, physical HSs (HS-PBEa and HS-PBEb) present spherical droplets randomly distributed in the bituminous phase, covering a surface fraction of roughly 8%, superior than that of the polymer concentration in the sample (3 wt %). This fact confirms the polymer swelling by maltenic oils and reveals their partial compatibility, which is required to improve the performance [11]. Thus, for physical HSs, a multiphasic structure is formed by two dispersed phases (non-dissolved CTR particles and polymer-rich droplets) within a continuous bituminous phase. Therefore, the synergistic contribution of these phases would explain the reported rheological and technological results [10,21,22].

On the other hand, in the case of reactive HSs, even though a fluorescent dispersed polymer-rich phase is also recognisable, the morphologies are deeply different. Thus, the surface fractions of the light regions are clearly below the proportion of added polymers (3 wt % for HS-EMAs and 2 wt % for HS-RPO), and microphases present smaller sizes than those of physical HSs. It is well known that if a polymer is physically dissolved in the bituminous matrix, then its original structure is lost during mixing and the macroscopic properties would not be transferred to the modified bitumen [13]. According to this, the observed morphologies of reactive HSs cannot explain the strong variation of their rheological behaviour. Therefore, although the presence of both polymer-droplets and non-dissolved CTR particles (along with the dissolved rubber chains) would partially contribute to enhance the rheological behaviour, the main origin of the modification seems to be caused by chemical changes that happens at a much lower scale. Thus, new chemical structures, undetectable by optical microscopy, are developed in reactive HSs by forming chemical bonds between polymer molecules and some bituminous compounds.

Regarding HS-EMAs, since these samples were formulated using a maleated copolymer, the reported behaviour can be explained on the basis of the chemistry of reactive pendant groups (free succinic anhydride). As reported in the bibliography, cyclic anhydrides can easily react with hydroxyl and amine groups giving rise to ester and amide bonds, respectively, in a ring opening reaction [23]. Therefore, as such groups are abundant in bitumen [24], succinylation reactions are expected to happen, linking copolymer chains covalently to some polar bituminous compounds (resins and asphaltenes). Therefore, new complex chemical structures are developed, leading to structural arrangements at a molecular scale and conferring the reported enhancement of in-service properties. According to this, the observed critical gel-like behaviour is then attributed to the formation of a chemical network. This assumption is consistent with the copolymer composition and it would explain why EMAa is a more effective modifier than EMAb. Thus, the higher proportion of succinic grafted groups in EMAa leads to a superior reactivity and, therefore, modification capacity, despite presenting a higher MFR i.e., lower polymer melt viscosity (Table 1) [15].

Finally, regarding the reactive polyolefin HS-RPO, again, the improvement should be attributed to chemical interactions between RPO and bitumen compounds [14,25]. In fact, it has been reported that the presence of grafted glycidyl methacrylate groups in olefin copolymers exerts a compatibilizing role with certain bitumen molecules [26,27]. Therefore, according to the epoxy chemistry, it is believed that oxiranic rings react with polar nucleophilic bitumen molecules, allowing an improvement of the miscibility between them, a fact that would explain the reported morphology [25]. Thus, as it has been reported in the bibliography, a ring-open reaction happens between the epoxy group and functional carboxylic acid or hydroxyls groups of polar molecules in bitumen to form ester or ether bonds, respectively, leading to formation of a network [27].

Taking into account that complex bituminous molecules are grouped forming a micellar structure containing more than one nucleophilic group, a chemical network structure may be built-up by reaction with the grafted groups of the olefinic chains of EMAs and RPO. Therefore, the consolidation of the three-dimensional structure would explain the reported critical gel behaviour and the improved hot storage stability. Thus, the formed network would interact with the non-digested CTR particles probably by entrapping them by physical means, hindering the settling of particles [10].

### 3.4. Correlation of Properties with Microstructure: MDSC Characterisation

MDSC tests were performed to provide structural information about crystalline and amorphous phases of the polymers and their compatibility and interaction with bitumen compounds [11]. Total heat flow curves (THF) of pristine PBEs, presented in Figure 1, point out a similar qualitative pattern characteristic of semi-crystalline ethylene/propylene copolymers, showing a clear glass transition (at −24.1 for PBEa and −27.7 °C for PBEb) followed by a cold crystallization and a single melting process (see Table 5).

Surprisingly, despite its high polypropylene content (Table 1), the melting pattern deviates from that of this homopolymer (with a melting process at around 163 °C) showing melting endothermal peaks at much lower temperatures. This result is attributed to the inclusion of ethylene comonomer in propylene backbone that interferes the crystallization process and shifts the melting temperature to lower values, [28].

**Figure 9:**
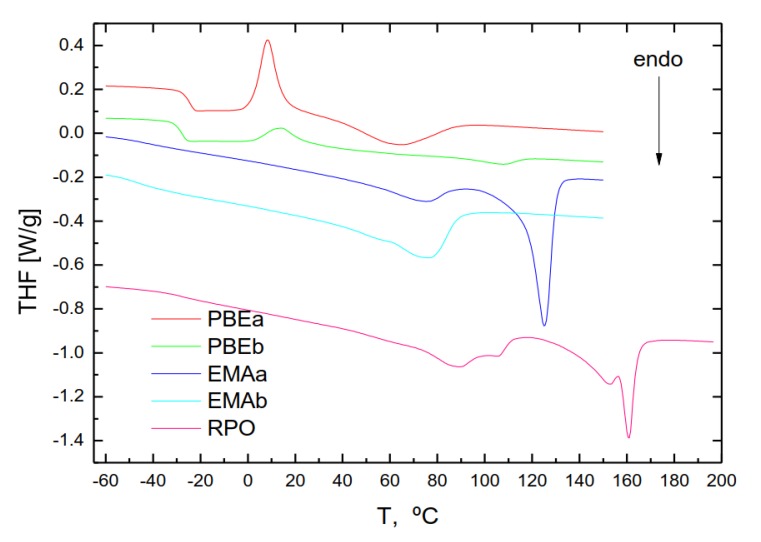
Total heat flow thermograms for neat polymers obtained from MDSC tests.

Regarding EMA copolymers, Figure 9 shows the presence of one (EMAb) or two (EMAa) melting peaks. The origin of this distinct behaviour cannot be precisely determined based solely on DSC results, because more experimental techniques are necessary to determine the nature of crystalline phases. However, according to the bibliography, the presence of an extra melting peak may happen in commercial ethylene-octene and ethylene-butene copolymers, reflecting two crystal populations with different thermal stability, and could be indicative of the presence of polymer fractions with different comonomer content in EMAa [29].

Finally, since RPO is a complex reactive polyolefin mixture, its DCS thermogram shows a multiple-peak pattern corresponding to the melting process of the crystalline fractions of its constituents, isotactic polypropylene, ethylene vinyl acetate copolymer and polyethylene wax.

**Figure 10:**
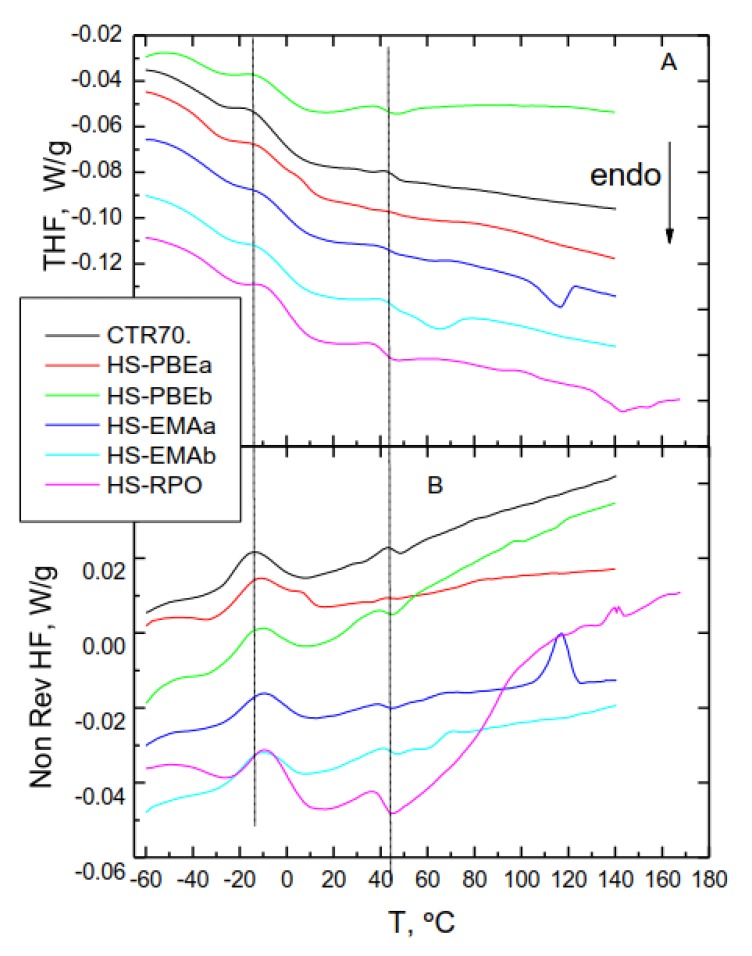
(**A**) Total heat flow thermograms and (**B**) non-reversing heat flow curves for HSs and CTR70, obtained from MDSC tests.

By contrast, total heat flow curves of the HSs presented in Figure 10A, display multiple thermal events resulting from both bituminous and polymer-rich phases. Firstly, by comparing THF curves of neat polymers and their corresponding HSs, a compatibility analysis of the crystalline fraction with bitumen can be performed. The degree of crystallinity of the polymer phase was calculated from the melting enthalpies of HSs weighted by its mass fraction, divided by 293 J/g for the heat of fusion of 100% crystalline polyethylene (Table 5). In the case of non-reactive polymers, Table 5 and Figure 10 point out a lowering of the melting point (8.3 °C for PBEa and 6.7 °C for PBEb) and a reduction of crystallinity, due to the migration of maltenic molecules to the polymer crystalline regions. However, taking into account their low crystallinity (compared to the homopolymers), this result does not completely explain the reported enhancement in the rheological behaviour of HS-PBEs since conventional polyolefins usually show poor compatibility with bitumen. In fact, the metallocene catalysis polymerization method used for producing PBEs, seems to be behind the moderate compatibility that allows the interdiffusion phenomena that leads to the swollen polymer-rich phase observed in Figure 8A,B. Thus, the structure of these metallocene-based polyolefins (polydispersity and degree of short-chain branching) favours a better dispersion in the bituminous matrix and improves the in-service properties [26,30].

On the other hand, the used reactive polymers and their corresponding modified bitumens display a much higher crystalline fraction than PBEs and HSs (HS-EMAa, HS-EMAb and HS-RPO). Similarly, these systems are characterised by a melting point depression and small reduction in χ_c_ as well. In spite of this, the observed micromorphologies (Figure 8C–E) does not seem to reflect this outcome because of the small proportion of light toned regions. However, as micrographs show a lower population of smaller fluorescence drops, this result hints at lower crystallite sizes and improved dispersion of the copolymer. This result, together with the improved reactive compatibility, suggests that the chemical network will also include submicron crystalline domains via covalent chemical bridges between macromolecules. Consequently, this more complex morphology, invisible by optical microscope, would be responsible for the enhancement in the thermomechanical behaviour.

On the other hand, total heat flow (major events) and non-reversing heat flow (minor transitions) curves in Figure 10 disclose changes induced in the rubberised bitumen microstructure by the addition of the polyolefin copolymer.

Thus, total heat flow curves of HSs result from the contribution of the polymer-rich phase, bituminous phase and remaining CTR particles [11]. As it was previously discussed, the polymer-rich phase causes the endothermic melting events. By contrast, rubberised bitumen presents several overlapping thermal effects as a consequence of the developed structures, due to time-dependent ordering processes that happen when bitumen is cooled down from the molten state [3,10,30]. These shuttle transitions can be analysed in a better way by using the non-reversing component of heat flow (Figure 10B) [11,30]. First of all, the broad endothermic background observed from −50 to 100 °C, has been mainly attributed to the melting of ordered mesophasic structures developed by simple aromatic bituminous compounds and, in a lower degree, to crystallized saturates. In addition, low and high molecular weight segments of saturated and aromatic compounds undergo the two cold crystallization peaks placed at around −15 and 40 °C, respectively. Additional cold crystallization exothermic peaks, associated to polymer phases, appear for some samples: HS-PBEa (6 °C) and HS-EMAa and b (~117 and 71 °C respectively). Furthermore, a minor endotherm can be viewed approximately at around 50 °C, is associated to larger mesophasic structures, and is found in resin and asphaltene fractions. Although the complex nature of HSs makes difficult a structural analysis, in general, as all these events undergo a modification in their intensities and positions, it can be interpreted as a proof of interactions between bitumen molecules and the added polymer [3,10,11,26].

This study has shown that the idea of combining CTR with a second polymeric phase to formulate the so-called Hybrid Systems (HSs) seems to be a proper alternative to achieve hot storage stability and to improve binder quality. Therefore, the resulting pavement is expected to present a higher resistance to rutting and thermal cracking and a reduced sensibility to temperature changes.

Non-reactive HSs (HS-PBEa and HS-PBEb), which were formulated with two semi-crystalline ethylene/propylene based elastomers, yield stable binders with improved in-service properties without deteriorating low temperature flexibility.

Reactive HSs (HS-EMAa, HS-EMAb and HS-RPO) use ethylene-based polymers containing functional groups (pendant succinic anhydride in EMAa and EMAb, and epoxy groups in RPO) that react with bitumen polar molecules, leading to a much higher degree of improvement than PBEs.

From a structural point of view, the enhancement in the thermomechanical response is a consequence of the development of a complex multiphasic system formed by non-dissolved CTR particles, a swollen polymer-rich phase and a bituminous phase. Physicochemical interactions and the synergistic effect among these phases contribute to the improved bulk properties, and favour heat storage stability.

Finally, the obtained results allow us to rank samples with respect to the capability of modification of the ethylene polymer as follows: PBEb ≈ PBEa << EMAb < EMAa < RPO.

## Figures and Tables

**Table 1:** Melt flow rates, at 230 °C, and polymer composition of the ethylene-based polymers used.

Name	Description	MFR ^(A)^	Composition
PBEa **^(B)^**	Ethylene-propylene elastomer	8	Ethylene 11.0 wt %
PBEb	Ethylene-propylene elastomer	20	Ethylene 15.0 wt %
EMAa	ethylene-co-1-octene copolymer functionalized with maleic anhydride	17	Maleic Anhydride 0.85% wt %
EMAb **^(C)^**	ethylene-co-1-octene copolymer functionalized with maleic anhydride	8	Maleic Anhydride 0.35 wt % Octene: (28 wt %)
RPO	Mixture: Isotactic polypropylene, ethylene-vinyl acetate (EVA), polyethylene wax and poly glycidylmethacrylate	-	Poly(glycidyl methacrylate) 5 wt %

^**(A)**^ MFR: Melt flow rate at 230 °C (g/10 min) ISO1133; ^**(B)**^ PBEpropylene-based elastomer; ^**(C)**^ EMA Ethylene copolymer functionalized with Maleic Anhydride

**Table 2:** Devices employed, normative and technological tests performed on bituminous samples.

Test	Standard	Device
Penetration	UNE-EN 1426	Petrotest PNR12 (Germany)
Softening point	UNE-EN 1427	Herzog Walter GmbH (Germany)
Penetration Index	UNE-EN 12591	(calculated from Penetration and Softening point)
Force ductility test at 5 °C	UNE-EN 13589	Mecánica Científica (Spain)
Elastic Recovery at 25 °C	UNE-EN 13398	Mecánica Científica (Spain)
Fraass breaking point	UNE-EN 13399	Petrotest BPA 5 (Germany)
Polymers dispersion on bitumen. Microscopy	UNE-EN 13632	Leica DM 2500 (Germany)
Storage stability at 180 °C	UNE-EN 13399	Toothpaste tubes
Multi Stress Creep Recovery (MSCR)	UNE-EN 16659	DSR Rheometer 302, Anton Paar (Austria)

**Table 3:** Selected normative properties for the bituminous binders studied.

Property	Unit	HS-PBEa	HS-PBEb	HS-EMAa	HS-EMAb	HS-RPO	CTR 70	70/100
Penetration	0.1 mm	60	61	63	63	54	70	75
Softening Point, T_R&B_	°C	56	56	68	63	63	51	46
Penetration Index	-	0.6	0.6	3.2	2.3	0.5	0.1	−1.3
Fraass Point	°C	−18	−25	−21	−19	−20	−20	−10
Elastic Recovery at 25 °C	%	64	61	62	61	60	61	13
Dif. of T_R&B_ (Stability) ^(A)^	°C	2.1	4.3	1.4	7.5	1.2	10	0
Cohesive Energy	J/cm^2^	3.088	3.636	3.923	3.075	2.019	2.702	0.007

^(A)^ Difference between softening point of the top and the bottom sections of the stability tube.

**Table 4:** Non-recoverable creep compliance (*J*_nr_), the percent of elastic recovery (%*R*) and minimum %*R* obtained from MSCR tests.

	Non-Recoverable Creep Compliance *J*_nr_ (kPa^−1^)		% Recovery (%*R*)		Minimum %*R*_3.2 kPa_ =$29.37J_{nr\;3.2\;\mathrm{kPa}}^{-0.263}$
	0.1 kPa	3.2 kPa	0.1 kPa	3.2 kPa	3.2 kPa
**HS-PBEa**	0.510	0.718	39.6	21.3	32.0
**HS-PBEb**	0.216	0.709	58.4	18.5	32.2
**HS-EMAa**	0.112	0.415	83.8	44.3	37.0
**HS-EMAb**	0.275	0.613	64.8	28.9	33.4
**HS-RPO**	0.360	0.767	60.0	35.1	31.5
**70/100**	4.720	5.071	0.7	0	-
**CTR-70**	0.976	1.398	30.7	10.6	26.9

**Table 5:** Enthalpies and melting temperatures and calculated crystalline fraction (*χ*_c_) obtained from modulated differential scanning calorimetry (MDSC) tests.

Samples	*T*_m_ (°C)	Δ*H*_m_ (J/g)	*χ*_c_ (%)
PBEa	64.1	14.2	4.8
HS-PBEa	55.8	0.30	3.4
PBEb	107.5	2.79	1.0
HS-PBEb	100.8	0.0113	0.1
EMAa	75.2/125.3	47.2	16.1
HS-EMAa	116.7	1.19	13.5
EMAb	75.9	75.9	25.9
HS-EMAb	1.73	64.2	19.7
RPO	89.5/105.0/ 153.5/160.0	51.6	17.6
HS-RPO (2%)	52.0/98.8/106/143	0.94	16.0

## Data Availability

The raw/processed data required to reproduce these findings cannot be shared at this time due to technical or time limitations.
